# Intracellular signaling entropy can be a biomarker for predicting the development of cervical intraepithelial neoplasia

**DOI:** 10.1371/journal.pone.0176353

**Published:** 2017-04-28

**Authors:** Masakazu Sato, Kei Kawana, Katsuyuki Adachi, Asaha Fujimoto, Mitsuyo Yoshida, Hiroe Nakamura, Haruka Nishida, Tomoko Inoue, Ayumi Taguchi, Juri Ogishima, Satoko Eguchi, Aki Yamashita, Kensuke Tomio, Osamu Wada-Hiraike, Katsutoshi Oda, Takeshi Nagamatsu, Yutaka Osuga, Tomoyuki Fujii

**Affiliations:** 1 Department of Obstetrics and Gynecology, Graduate School of Medicine, The University of Tokyo, Bunkyo-ku, Tokyo, Japan; 2 Department of Obstetrics and Gynecology, School of Medicine, Nihon University, Itabashi-ku, Tokyo, Japan; Fondazione IRCCS Istituto Nazionale dei Tumori, ITALY

## Abstract

While the mortality rates for cervical cancer have been drastically reduced after the introduction of the Pap smear test, it still is one of the leading causes of death in women worldwide. Additionally, studies that appropriately evaluate the risk of developing cervical lesions are needed. Therefore, we investigated whether intracellular signaling entropy, which is measured with microarray data, could be useful for predicting the risks of developing cervical lesions. We used three datasets, GSE63514 (histology), GSE27678 (cytology) and GSE75132 (cytology, a prospective study). From the data in GSE63514, the entropy rate was significantly increased with disease progression (normal < cervical intraepithelial neoplasia, CIN < cancer) (Kruskal-Wallis test, p < 0.0001). From the data in GSE27678, similar results (normal < low-grade squamous intraepithelial lesions, LSILs < high-grade squamous intraepithelial lesions, HSILs ≤ cancer) were obtained (Kruskal-Wallis test, p < 0.001). From the data in GSE75132, the entropy rate tended to be higher in the HPV-persistent groups than the HPV-negative group. The group that was destined to progress to CIN 3 or higher had a tendency to have a higher entropy rate than the HPV16-positive without progression group. In conclusion, signaling entropy was suggested to be different for different lesion statuses and could be a useful biomarker for predicting the development of cervical intraepithelial neoplasia.

## Introduction

While the mortality rates of cervical cancer have been drastically reduced since the introduction of the Pap smear test, it remains one of the leading causes of death in women worldwide [1]. The defining characteristic of cervical cancer is that its initiator is viral infection or infection with human papilloma virus (HPV) in cervical epithelia [1–3]. HPV infection is a transient infection in most patients; however, persistent infection can occur in some patients, resulting in progression of the lesion to cervical dysplasia, carcinoma *in situ* and invasive cancer. Cervical lesions are classified into cervical intraepithelial neoplasia (CIN) 1, 2 and 3 based on its relationship with the prognosis. CIN 1 is mild dysplasia, which is mostly observed because it disappears as part of its natural course. CIN 3 includes severe dysplasia and carcinoma *in situ*, and management involves treatment because it is highly likely to develop into invasive cancer. CIN 2 is moderate dysplasia, and the choice of whether to observe or treat the patient depends on the patient’s condition and wishes. Generally, conization, which is one of the most common treatments for cervical lesions, increases the risks of postoperative complications and pregnancy complications, such as abortion and preterm labor [4, 5]. Thus, the treatment decision should be carefully made. On this topic, many studies have been performed to appropriately evaluate the risk of developing cervical lesions [2, 3, 6]. Although detection of high-risk HPV has a high negative predictive value, its positive predictive value should be discussed because of the aforementioned transient infection.

Meanwhile, there have been many investigations on the diversity or heterogeneity of various solid tumor types [7, 8]. The term, diversity, has several views. There can be mixtures of cells that are resistant or sensitive to chemotherapy in the same tumor, mixtures of cells with different metabolic profiles, and the coexistence of cells with different oxygen levels. The same is true for gene expression and networks in each cell in the same tumor. In other words, gene expression network patterns are known to be more complicated among cancer cells and tumors than among normal cells and organs [9–14]. One group reported the evaluation of the complexity of gene networks by quantifying them from 0 to 1 as ‘signaling entropy’ using the data obtained from microarray analysis and RNA sequencing [9]. In the literature, signaling entropy has been found to be significantly higher in cancer cells, especially cancer stem cells, than in normal cells, thereby helping to distinguish them. Furthermore, studies have also indicated that measurements of signaling entropy can be markers for prognosis prediction in lung and breast cancer [10].

Taken together, we aimed to investigate whether measuring signaling entropy was useful for both distinguishing cervical cancer cells from normal cells and predicting the risk of developing cervical precancerous lesions. In other words, we hypothesized that signaling entropy in cervical lesions would increase with the disease progression. In addition, we also hypothesized that signaling entropy should help discriminate the pathologically normal cells if their prognoses differ.

Indeed, we confirmed that the signaling entropy increased with disease progression. Additionally, we found that the signaling entropy increased with HPV infection even in cells that were pathologically normal.

In the present study, we investigated whether measuring the signaling entropy can be useful for predicting the risks of developing cervical lesions. Further investigation is needed to determine how signaling entropy changes in each patient; however, signaling entropy was suggested to be a useful biomarker for predicting the development of cervical intraepithelial neoplasia.

## Materials and methods

### Measurement of signaling entropy

In this study, a Protein Interaction Network (PIN) was constructed, and the signaling entropy (SR) was measured as described in the literature [9, 10]. Briefly, studies have described that protein interactions in the network include physical stable interactions such as those defining protein complexes, as well as transient interactions such as post- translational modifications and enzymatic reactions found in signal transduction pathways. The R-scripts for the computation of signaling entropy were uploaded and are available for download at www.sourceforge.net/projects/signalentropy [10]. The program in the present study was run by R (version 3.2.4). The expression profiles of probes mapping to the same Entrez gene IDs were averaged. as previously described [9, 10]. If RMA-normalized data were provided, this was used. If normalized data were not provided, raw data were downloaded and RMA-normalized [9, 10].

### Dataset

The datasets used in this study were GSE63514, GSE27678 and GSE75132 [1, 15, 16]. We used ArrayExpress to find datasets on cervical precancerous lesions. Among them, we decided to include GSE63514, GSE75132 and GSE27678 for the following reasons: GSE63514 included data from a laser capture microdissection experiment, GSE75132 included data from a prospective study, and GSE27678 contained data from a relatively large number of samples.

GSE63514 contained 128 samples (24 normal, 14 CIN1 lesions, 22 CIN2 lesions, 40 CIN3 lesions, and 28 cancers specimens). The platform used was an Affymetrix Human Genome U133 Plus 2.0 Array. The data were already RMA-normalized, and 19151 genes were annotated to Entrez Gene ID. Among them, 8127 genes were included in the PIN in the program.

GSE27678 contained 77 samples (15 normal, 11 low-grade squamous intraepithelial lesions, 21 high-grade squamous intraepithelial lesions, 28 squamous cell carcinomas, and 2 cell lines). The platforms used were an Affymetrix Human Genome U133 Plus 2.0 Array and Affymetrix Human Genome U133A 2.0 Array. The data were already RMA-normalized, and 12460 genes in common were annotated to Entrez Gene ID. Among them, 6932 genes were included in the PIN in the program.

GSE75132 contained 41 samples (11 HPV-negative, 20 HPV16-persistent without progression, and 10 HPV16-persistent with progression). The platform used was an Affymetrix Human Genome U133 Plus 2.0 Array. The data were not normalized, and RMA-normalization was performed. Here, 19150 genes were annotated to Entrez Gene ID. Among them, 8127 genes were included in the PIN in the program.

### Statistical analysis

For statistical analysis, JMP Pro 11(SAS, USA) was used, and *p value*s less than 0.05 were considered statistically significant. Wilcoxon rank-sum test was used for comparing medians between two groups. Kruskal-Wallis test was used for comparing more than two groups.

## Results

### Entropy rate increases along with the progression of cervical lesions in histological specimens

GSE63514 was from the Study to Understand Cervical Cancer Early Endpoints and Determinants (SUCCEED study), and it contained 128 samples [1]. Fresh-frozen cervical samples were cryosectioned and histopathologically evaluated. A Laser.

**Fig 1:**
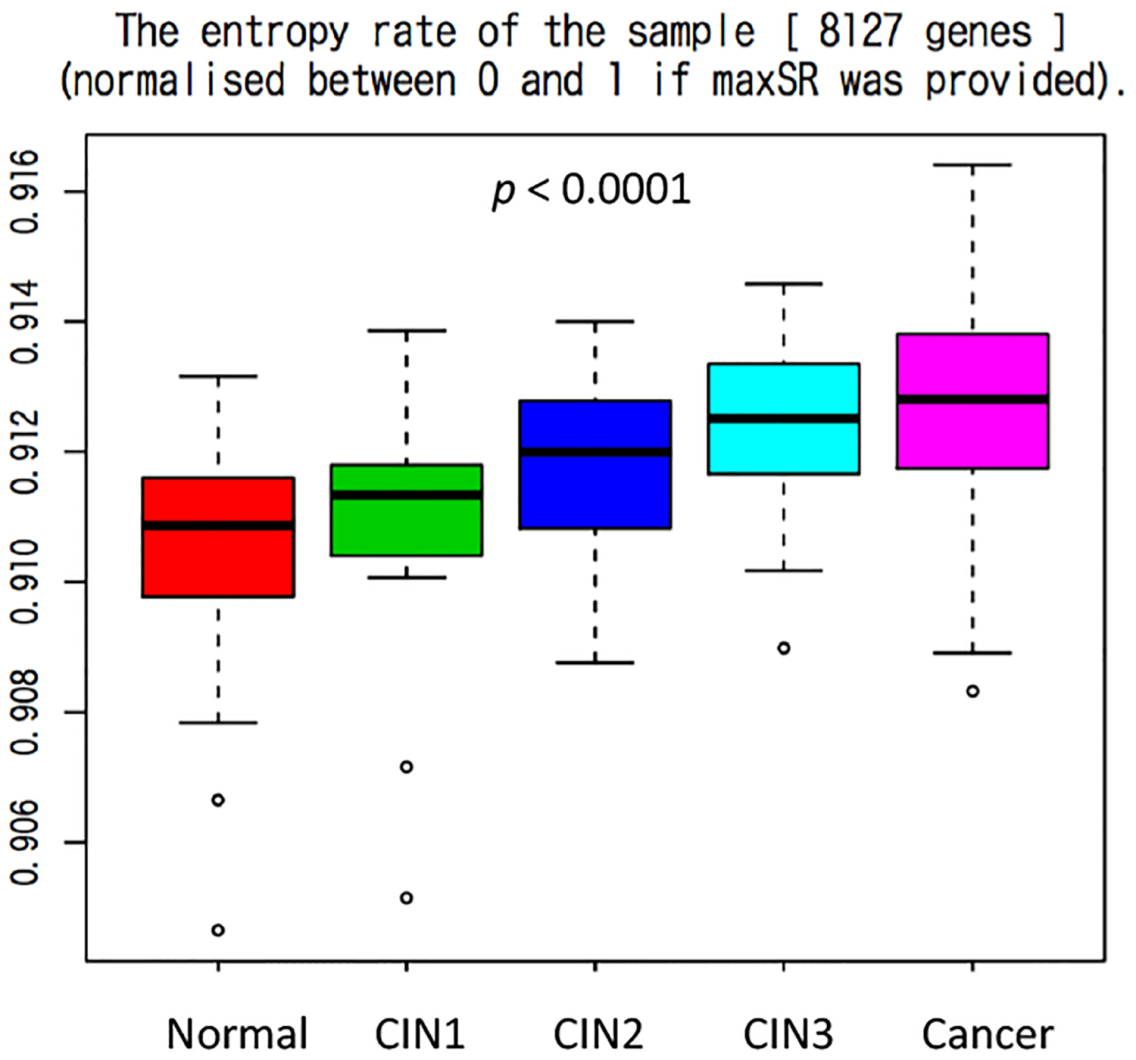
Measurement of the signaling entropy in GSE63514. The entropy rate was significantly higher according to the disease progression (Kruskal-Wallis test, *p* < 0.0001). Cancer; invasive cancer.

Capture Microdissection System was then used to capture the epithelial lining of the cervix from normal healthy control specimens or precancerous and invasive cancerous cell masses from cervical lesions. Afterwards, the specimens were processed for comprehensive human mRNA-level measurements. As shown in Fig 1, the entropy rate significantly increased according to the progression of the cervical lesion (Kruskal-Wallis test, p < 0.0001).

### Entropy rate increases along with the progression of cervical lesions in cytological specimens

**Fig 2:**
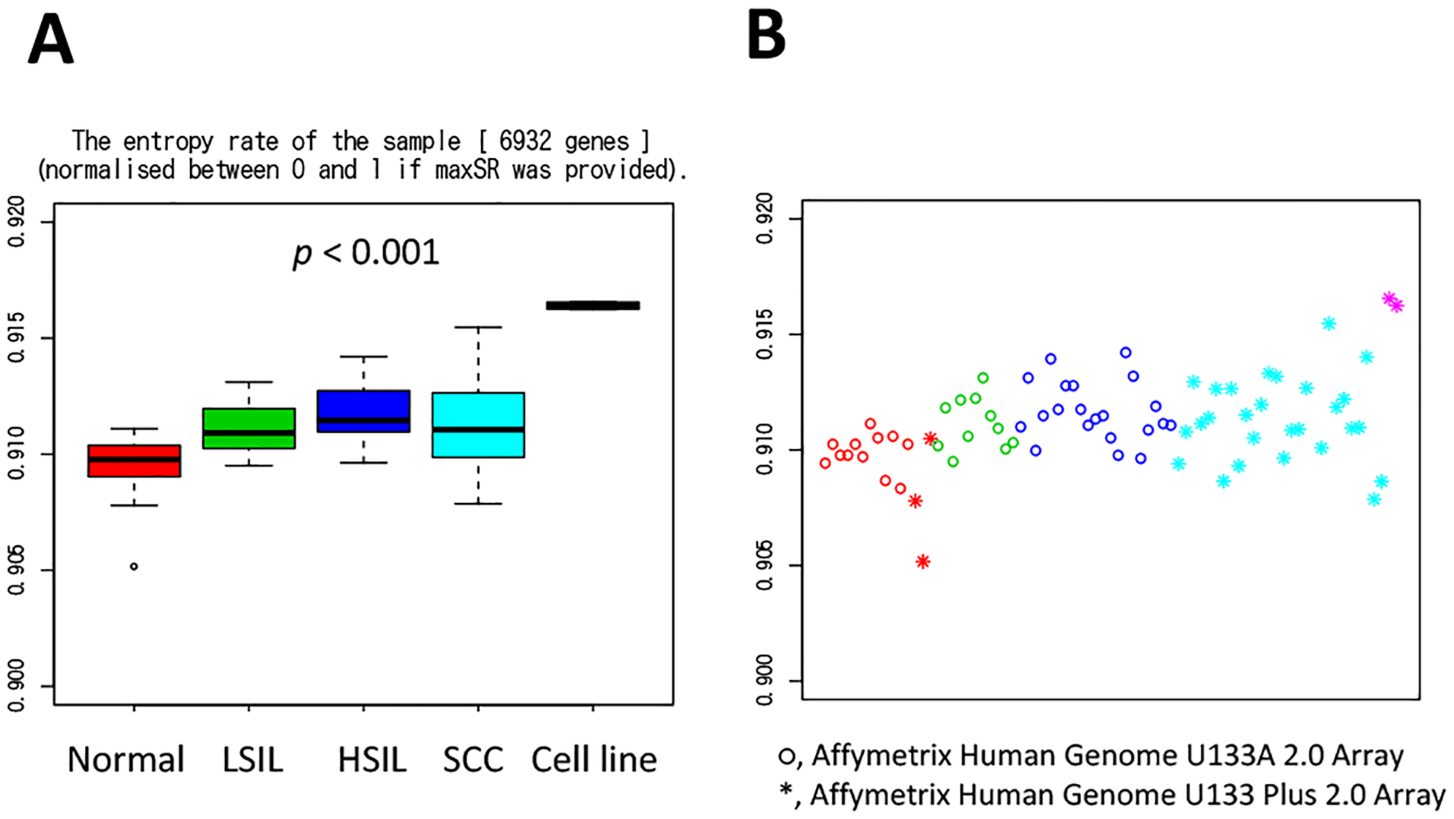
Measurement of the signaling entropy in GSE27678. (A) Boxplot of the entropy rate from whole samples. The entropy rate increased according to the disease progression (Kruskal-Wallis test, *p* < 0. 001). Among them, the entropy rate from the cell line was highest, as described in the literature [9]. (B) Entropy rate from each platform. ○ represents the data obtained from the Affymetrix Human Genome U133A 2.0 Array, and * represents the data obtained from the Affymetrix Human Genome U133 Plus 2.0 Array. The distribution patterns of the entropy rate seemed slightly different among the platforms.

We then investigated whether the above results could be applied to the data from cytological specimens. Cytology tests are used for screening, and, as a result, low-grade squamous intraepithelial lesions (LSILs) are likely to match CIN 1 in biopsies and high-grade squamous intraepithelial lesions (HSILs) are likely to match CIN 2–3 in biopsies. GSE27678 contained microarray data from 75 samples (15 normal, 11 LSIL, 21 HSIL, and 28 squamous cell carcinomas) and 2 cell lines (77 samples in total) [16]. The platforms used were an Affymetrix Human Genome U133 Plus 2.0 Array and Affymetrix Human Genome U133A 2.0 Array. As shown in Fig 2A, the entropy rate significantly increased according to the disease progression (Kruskal-Wallis test, p < 0.001). In addition, as shown in the literature, the entropy rate from the cell line was the highest among them. The entropy rate from squamous cell carcinoma (SCC) was slightly lower than that from HSILs, but the influence of the different platforms on the distribution patterns of the entropy rate could not be excluded (Fig 2B).

### Entropy rate is higher in the HPV-persistent groups than the HPV-negative group

**Fig 3:**
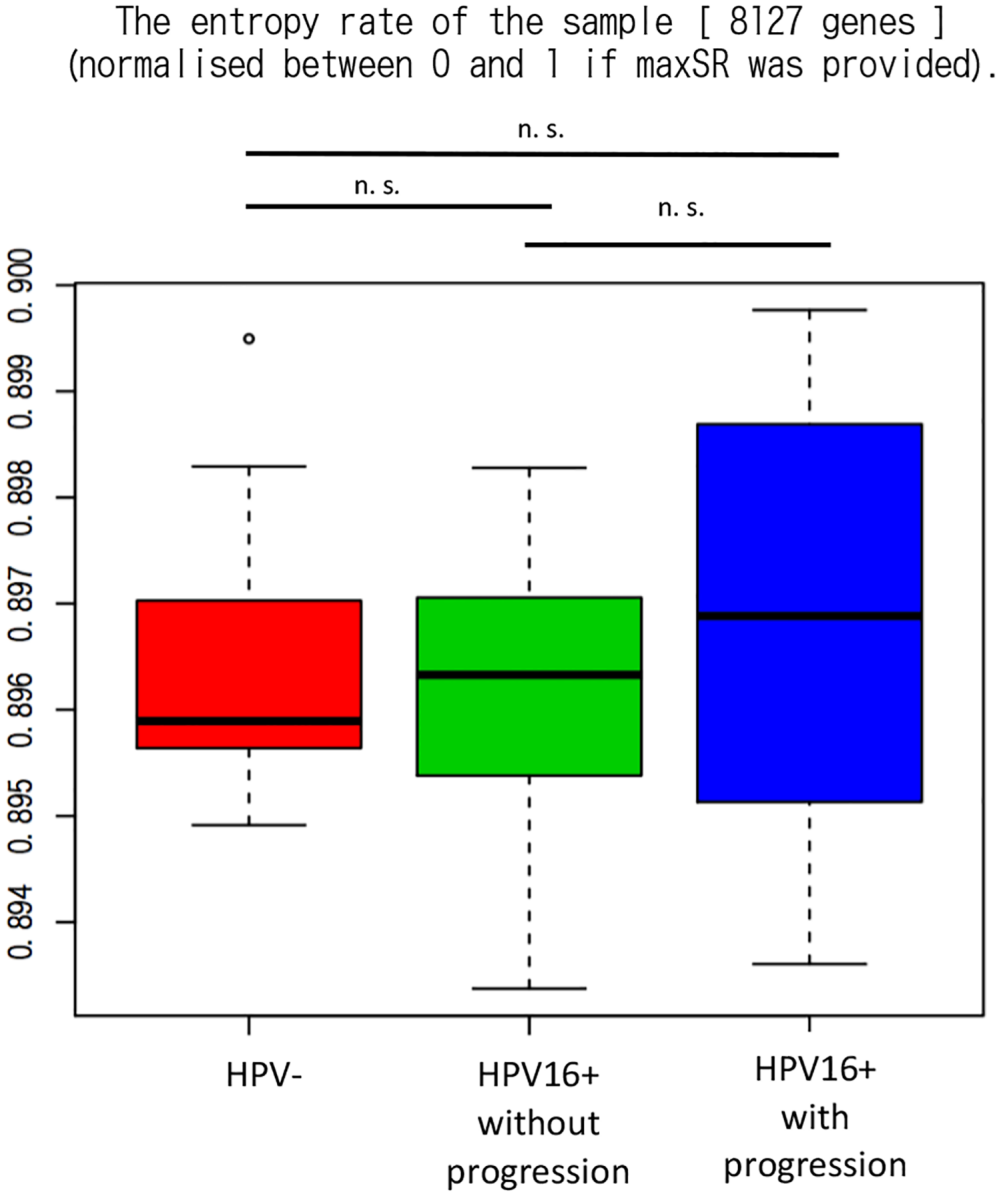
Measurement of the signaling entropy in GSE75132. The median of the entropy rate was higher in the HPV16-persistent (HPV16+) groups than the HPV-negative (HPV-) group. The group that was destined to progress to CIN3+, or the HPV16-persistent with progression group, tended to have a higher entropy rate than the HPV16-persistent without progression group.

Thus far, we observed the distribution patterns of the entropy rate in lesions. We then investigated whether the entropy rate could be a biomarker for predicting the prognosis of cervical lesions. GSE75132 was a dataset from a prospective cohort study [15]. Enrolled women visited the hospital twice, two years apart (visits 1 and 2). Among women with normal cytology at visit 2, those who had HPV16 at both visits 1 and 2 were assigned to the HPV16-persistent groups, while those who did not have HPV at both visits 1 and 2 were assigned to the HPV-negative group after age-matching. Furthermore, among the HPV16-persistent patients, those who progressed to CIN3+ were defined as the HPV16-persistent with progression group, and the others were classified as the HPV16-persistent without progression group with as many as 19.3 follow-up periods. CIN3+ included severe dysplasia, carcinoma *in situ* (including adenocarcinoma *in situ*), CIN3 or cancer (including adenocarcinoma). Finally, the dataset contained 41 samples (11 HPV-negative, 20 HPV16-persistent without progression, and 10 HPV16-persistent with progression). The platform used was an Affymetrix Human Genome U133 Plus 2.0 Array. As shown in Fig 3, the median of the entropy rate was higher in the HPV16-persistent groups than in the HPV-negative group. The group that was destined to progress to CIN3+, or the HPV16-persistent with progression group, especially tended to have a higher entropy rate than the HPV16-persistent without progression group. Thus, the signaling entropy was shown to be able to be a useful biomarker for predicting the development of cervical intraepithelial neoplasia.

## Discussion

In the present study, we showed that the signaling entropy could be useful for predicting the prognosis of cervical epithelia.

Cervical cancer is one of the leading causes of death in women worldwide [1]. The cause of cervical cancer is viral infection or infection with human papilloma virus (HPV) in cervical epithelia [1–3]. HPV infection is mostly a transient infection; however, it can be a persistent infection in some patients, resulting in progression of the lesion to cervical dysplasia, carcinoma *in situ* and invasive cancer. Therefore, many researchers have investigated how to appropriately evaluate the risk of developing cervical lesions. We sought to detect high-risk cases by focusing on signaling entropy.

There are multiple reasons why we focused on signaling entropy, especially in cervical cancer. In the literature, authors have proposed a measure of signaling pathway promiscuity as an approach for quantifying the stemness and heterogeneity of any given cancer sample [9]. In other words, they based the concept of cancer stem cells (CSCs) on measuring the signaling entropy. CSCs are a small population of cells within a tumor that are thought to be related to tumorigenesis and tumor invasiveness [7, 8]. The group demonstrated that the signaling entropy was significantly higher in cancer cells, especially CSCs, than in normal cells [9]. The origin of CSCs remains controversial; however, the hypothesis that CSCs originate from normal stem cells is an acceptable one [17–21]. In the context of cervical carcinogenesis, cervical epithelial stem (progenitor) cells, or reserve cells, are known to acquire malignancy after being infected by HPV, and they are forced to continuously express HPV-derived oncoproteins E6 and E7 [17]. Cervical CSCs have yet to be pathologically quantified. Taken together with the consideration that CSCs should probably exist among pathological samples that we call precancerous lesions, we speculated that the signaling entropy could provide insight into the development of cervical lesions. Furthermore, we focused on cervical cancer because it was expected that investigating precancerous lesions in the cervix might provide other researchers with insights into different types of solid tumors. Additionally, the cervix is anatomically easy to biopsy and analyze by cytology, and it could be a good model for investigating the development of precancerous lesions.

Indeed, we confirmed that the signaling entropy increased along with the disease progression. Additionally, we found that the signaling entropy could help identify the prognosis of cells that were pathologically normal. In addition, the signaling entropy was shown to be able to help predict the prognosis of cervical epithelia.

There are two limitations in the present study. One is that the ranges of the signaling entropy (i.e., the y-axis in the figures) were different in each experiment. This is thought to be due to, at least in part, the differences in the experimental procedures, such as biopsies and cytologies, and the methods for collecting specimens in an individual facility, the numbers and kinds of cells collected, and the platforms used for the microarray. Therefore, we could not investigate cut-off values of the signaling entropy. The other limitation is that GSE63514 and GSE27678 were datasets from retrospective studies, and GSE75132 was a dataset in which the signaling entropy could only be measured at one point even though it was from a prospective study. In other words, we could not identify the time course of signaling entropy in each patient. For instance, we do not know whether a CIN 2 patient with a high signaling entropy rate is likely to progress to CIN 3 or if a CIN 2 patient with a low entropy rate is likely to regress to CIN 1. We also do not know whether the signaling entropy gradually increases or drastically increases (with the occurrence of CSCs, for instance). Signaling entropy is known to be more increased by inactivation of tumor suppressor genes than activation of oncogenes [14]. On the other hand, high-risk HPV integration (or episomal forms of high-risk HPV), which is known to be the cause of HPV E6 overexpression and results in the degradation of tumor suppressor proteins including p53, is involved in neoplastic progression in cervical cancer [22–24]. Collectively, the signaling entropy might increase according to HPV integration in cervical carcinogenesis. In any case, a prospective study on the above should be performed.

The strength of measuring the signaling entropy is that this approach comprehensively assesses the data. While omics data analysis enables us to obtain substantial information, it also detects variables of uncertain significance [25]. Thus, an appropriate reduction of the variables, without missing significance, is needed. To measure the signaling entropy, a PIN is first constructed [9,10]. This process pulls out biologically significant networks that are expected by gene expression patterns from among enormous and redundant networks. In other words, the PIN only contains 8434 nodes at maximum. Measuring the signaling entropy is not necessarily the absolute approach for variable reduction; however, an approach that comprehensively handles the data and simply obtains values is easy to understand and use, especially when people without training in bioinformatics are analyzing the data.

In the present study, we investigated whether measuring the signaling entropy can be useful for predicting the prognosis of cervical epithelia. Further investigation is needed to determine the time course of the signaling entropy in each patient; however, the signaling entropy was suggested to be a useful biomarker for predicting the development of cervical intraepithelial neoplasia.
